# Ovarian Tumors*From the Bistoury of April, 1871, and republished by request.

**Published:** 1879-01

**Authors:** 


### OVARIAN TUMORS. *

The operation for the removal of Ovarian Tumors has become so popular and is so frequently performed of late years, that almost every lady has learned something of it, and has formed some sort of an idea—generally erroneous—as to the nature of the tumor and the means of the cure.

In this article we hope to be able to explain what an ovarian tumor really is, and to give some timely advice to those suffering from such a difficulty.

It must be known that the ovaries are two organs, situated near and on either side of the uterus, within the substance of the *broad ligaments* which hold the uterus in position. They are about the size and shape of an almond, and contain a transparent fluid and the *ovum*, or egg, which, after having passed by the fallopian tubes into the uterus, and having become fecundated, grow and form the living fœtus. These ovaries often become diseased, increased in size, and secrete a highly albuminous fluid, until tumors of immense size are formed. The causes for these growths are generally obscure, the patient or physician being unable to give any satisfactory reason for their development. Inflammation of the ovaries, from difficult labor, provoked abortion, suppression of the catamenia, injuries from falls, or blows upon the abdomen, colds contracted during the catamenial periods, and the various chronic ailments of the uterus, are the known and usual causes for the formation of these tumors. There are several kinds of tumors that spring from the ovaries, among the most common of which are the fibrous, cartilaginous, and *encysted*. It is of the latter class that we desire to write at present. The encysted tumor of the ovary, so called from the fluid being contained in a *cyst* or sac, is formed by the deposit, within the ovary, (the walls of which gradually distend and enlarge,) of a thin, watery, straw-colored, or coffee-colored fluid, highly albuminous—it becoming almost solid when boiled. The symptoms accompanying the formation of such a tumor are not always the same in different individuals. Usually the first indication of their presence is a dull pain, low down in the abdomen, and to the right or left side. This pain soon subsides, then returns again with increased vigor; the catamenia becomes irregular, and finally ceases entirely; soon a round or oval tumor is found to the right or left side, and on a line with the top of the hip bones; it is usually soft, movable and elastic, the patient being able to thrust the fingers under it and to move it about in the abdomen; it gradually increases in size, *from below upwards*, and soon passes to the centre, and finally over the entire abdomen. Its growth is now more rapid, the abdomen becomes enormously enlarged, extending up to the breast bone and under the ribs, encroaching upon the space occupied by the lungs, and producing a sense of suffocation, the patient being unable to breathe freely, unless bolstered up in bed. Sometimes prostration is rapid, at other times the patient is able to attend to her household duties until she finally becomes exhausted from the great weight of the tumor. If the tumor is now tapped, great relief will be experienced from the discharge of its contents; usually this fluid is of the color and consistence before described, but sometimes it is of a darker color, and so thick that it cannot be discharged through an ordinary canula. Often, vast quantities of fluid are obtained, measuring many gallons, at other times very little is secured from a very large tumor, so that its size is not perceptibly diminished. When the latter is the result, it shows either that the contents are too thick to be discharged, that the tumor is solid, or that more than one cyst exists, and that the one containing most of the fluid lies too far back to be reached by the trocar. It will thus be seen how tapping is used as a means of diagnosis for the surgeon, as well as relief for the patient. But, as this relief is only temporary, tapping should not be persisted in. The sac will refill in from three to twelve weeks, and at every repeated tapping the fluid becomes thicker, the adhesions of the tumor to the abdominal walls greater, while the patient loses strength more rapidly. The time to be selected for the extirpation of the tumor depends greatly upon the condition of the patient. This can only be determined by the surgeon. As a general thing, the patient demands an early operation, in order to be relieved from her pain, while her friends are apt to extend the time until she becomes too much prostrated to withstand the shock consequent upon such an operation.

Usually we do not advise surgical interference as long as the patient is able to engage in her household duties, and is tolerably free from pain. But the moment she is confined to her room, and is constantly asking for relief from her burden, it should come promptly, through the skillful surgeon's knife.

Medicines, taken with a view to cure an ovarian tumor, are worse than useless. We frequently meet with cases that have been medicated until

* From the BISTOURY of April, 1871, and republished by request.

they are completely prostrated, months being necessary to restore them to sufficient health and strength to undergo an operation. No medicines should ever be taken save those that are intended to invigorate the general health. An ovarian tumor can never be cured by medicines, therefore avoid those traveling charlatans who advise differently, and who prescribe medicines for money and not for disease.

The operation for the removal of an ovarian tumor, called by the surgeon *ovariotomy*, is of American origin, having first been performed by Dr. Ephriam McDowell, of Kentucky, in December, 1809. The first operation was a success, else, perhaps, no surgeon would have been again found bold enough to enter into so hazardous an undertaking, at least not until our present facilities for doing surgery had been perfected. Great improvements have been made of late years, in the manner of performing ovariotomy, and in the instruments employed, until it is now considered quite a safe and proper undertaking. Of course, the chances for recovery after such a formidable operation, depends greatly upon the strength and determination of the patient, the extent of the attachment of the tumor, and the skill of the operator. The *size* of the tumor has little to do with the sucess of the operation. Usually the larger the tumor the less liability to *peritonitis*, or inflammation of the lining membrane of the abdomen, which is the chief danger to be feared as a result of ovariotomy.

Successful ovariotomists now-a-days, save three-fifths of their cases, among which are tumors of immense size and weight. Among eighteen removed by ourself, one weighed 85 pounds [more than remained, in weight, of the patient,] six weighed from 32 to 45 pounds each; the remainder weighing from six pounds upward. The recovery from an operation is usnally rapid, the patient generally being able to walk out in from twenty to forty days. No pain, of course, is experienced from such an operation, as the patient is kept under the influence of chloroform or æther until its completion. The time occupied depends upon the extent of attachments of the tumor to surrounding parts, the surgeon generally being able to complete his operation in from thirty to sixty minutes. After an operation, much depends upon careful nursing, and carrying out *thoroughly*, the directions of the surgeon. Cases are sometimes lost from the carelessness of those having care of the patients. No one should enter into an operation of this magnitude without determination to obey, strictly, the injunctions of the surgeon and the attending physician. After a patient has fully decided upon having an operation performed, relatives and friends should be careful not to discourage her by relating the unfavorable results of other operations that they may have heard of. Many foolish people take particular delight in harassing a sufferer in this manner. If they are unable to find a case of disastrous surgery anywhere in their neighborhood, they will manufacture one from their own prolific brains, for the purpose. The patient requires to be encouraged so that she may enter upon the operation with fortitude and a determination to get well. Patients who are so fortified almost always recover, and surgeons accept such courage on the part of their patients, as an excellent symptom for a favorable termination of the operation.

